# Haema-Uro-Chrome

**Published:** 1914-03

**Authors:** 


HAEMA-URO-CHROME. Further note. On page 388 of the January issue we reviewed an article on this subject by Theodore G. Davis, in the *California State Jour. of Med.* The color is developed by heating slowly to boiling with 10 per cent. of HCl, cooling and extracting with ether, in a flask, indican appearing above the hæma-uro-chrome, which is claimed to be diagnostic of cancer. We have verified this test in a case of gastric cancer, but the test is practically identical with that for "uroerythrin" or "urorosein," boiling the urine in a test tube and precipitating with Barium chlorid, whose insoluble sulphate and phosphate bring down the coloring matter. A still more convenient way is to boil in a tube and extract with chloroform, which carries the pigment to the bottom and shows the same sharp discrimination between the red pigment, whatever it may be called, and indican. Having used essentially the same test for many years, we are able to state with confidence that it is characteristic of cancer after the disease has caused metabolic disturbances, that it does not appear until the cancer has produced such disturbance and that the same or an apparently identical pigment may be encountered at times in various other conditions. It certainly indicates some grave disturbance of metabolism and usually is found in highly colored and quite acid (75 per cent. by direct titration and over) urines. The converse, namely, that high colored and highly acid urines usually give the test, is not true in our experience, unless the moderate degree of pink that can be precipitated with barium or extracted with chloroform, ether, etc., directly or after heating with mineral acid, indicates the same reaction in slight degree. We would be glad to have further light shed on this reaction.

